# Direct Measurements of Interfacial Photovoltage and Band Alignment in Perovskite Solar Cells Using Hard X-ray Photoelectron Spectroscopy

**DOI:** 10.1021/acsami.2c17527

**Published:** 2023-02-27

**Authors:** Sebastian Svanström, Alberto García Fernández, Tamara Sloboda, T. Jesper Jacobsson, Fuguo Zhang, Fredrik O. L. Johansson, Danilo Kühn, Denis Céolin, Jean-Pascal Rueff, Licheng Sun, Kerttu Aitola, Håkan Rensmo, Ute B. Cappel

**Affiliations:** † Condensed Matter Physics of Energy Materials, Division of X-ray Photon Science, Department of Physics and Astronomy, Uppsala University, Box 516, SE-751 20 Uppsala, Sweden; ‡ Division of Applied Physical Chemistry, Department of Chemistry, KTH - Royal Institute of Technology, SE-100 44 Stockholm, Sweden; § Institute of Photoelectronic Thin Film Devices and Technology, Key Laboratory of Photoelectronic Thin Film Devices and Technology of Tianjin, College of Electronic Information and Optical Engineering, Nankai University, 300350 Tianjin, China; ∥ Division of Organic Chemistry, Department of Chemistry, KTH - Royal Institute of Technology, SE-100 44 Stockholm, Sweden; ⊥ Institute for Methods and Instrumentation in Synchrotron Radiation Research FG-ISRR, Helmholtz-Zentrum Berlin für Materialien und Energie Albert-Einstein-Strasse 15, 12489 Berlin, Germany; # Institut für Physik und Astronomie, Universität Potsdam, Karl-Liebknecht-Strasse 24-25, 14476 Potsdam, Germany; ∇ Synchrotron SOLEIL, L′Orme des Merisiers, BP 48 St Aubin, 91192 Gif sur Yvette, France; ○ Laboratoire de Chimie Physique-Matière et Rayonnement, Sorbonne Université, CNRS, 75005 Paris, France; ◆ State Key Laboratory of Fine Chemicals, Institute of Artificial Photosynthesis, DUT−KTH Joint Education and Research Centre on Molecular Devices, Dalian University of Technology (DUT), 116024 Dalian, China; ¶ Center of Artificial Photosynthesis for Solar Fuels, School of Science, Westlake University, 310024 Hangzhou, China; †† New Energy Technologies Group, Department of Applied Physics, Aalto University School of Science, Box 15100, 00076 AALTO, Finland

**Keywords:** operando measurements, photoelectron spectroscopy, photovoltaics, semiconductor physics, experimental design, device design, lead halide perovskite, solar cell

## Abstract

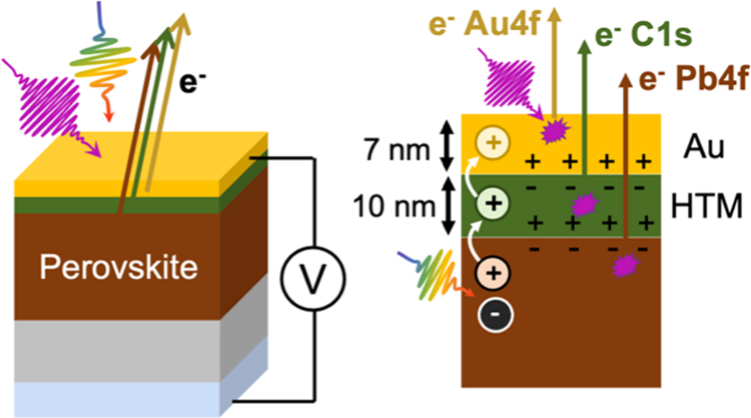

A heterojunction is the key junction for charge extraction in many thin film solar cell technologies. However, the structure and band alignment of the heterojunction in the operating device are often difficult to predict from calculations and, due to the complexity and narrow thickness of the interface, are difficult to measure directly. In this study, we demonstrate a technique for direct measurement of the band alignment and interfacial electric field variations of a fully functional lead halide perovskite solar cell structure under operating conditions using hard X-ray photoelectron spectroscopy (HAXPES). We describe the design considerations required in both the solar cell devices and the measurement setup and show results for the perovskite, hole transport, and gold layers at the back contact of the solar cell. For the investigated design, the HAXPES measurements suggest that 70% of the photovoltage was generated at this back contact, distributed rather equally between the hole transport material/gold interface and the perovskite/hole transport material interface. In addition, we were also able to reconstruct the band alignment at the back contact at equilibrium in the dark and at open circuit under illumination.

## Introduction

Solar cells are becoming an increasingly important part of our power production with silicon solar cells dominating the market. Thin film technologies like copper indium gallium selenide (CIGS), cadmium telluride (CdTe), and, more recently, lead halide perovskite solar cells have the potential to challenge the dominance of silicon. However, there are many uncertainties in the dynamics at the heterojunctions at the heart of these thin film devices,^1^ and such uncertainties are further complicated in lead halide perovskites by self-doping, ion migration, and trap formation linked to mobile defects in the material.^2^ Nonetheless, lead halide perovskites have achieved power conversion efficiency of over 25%, which is on par with silicon solar cells.^3^ Still, the commercialization of perovskite solar cells has been hindered by limited long-term stability, which has partially been linked to the dynamics at the interfaces of the device.^4^ These key interface regions are generally very thin, typically less than few nanometers, which makes them difficult to study.

Simulations can serve as a guidance to the design of more efficient solar cell devices by identifying suitable interface materials and parameters where optimization can be achieved.^5−7^ However, the models will need to accurately reproduce effects like charge trapping and recombination by mobile defects, which will require accurate estimates of the material properties, not only of the perovskite but also of the selective contacts and electrodes. It is important to obtain experimental insight into the interfacial energetics and its structure–function relationships to support such a modeling. This has largely been done using three techniques: Kelvin probe force microscopy (KPFM), electron beam-induced current (EBIC), and photoelectron spectroscopy (PES). KPFM is an imaging technique that allows the measurement of the potential difference of a material and the tip of a probe, giving a map of electrical potential. EBIC, on the other hand, measures the current induced by an electron beam, essentially emulating an extremely small beam of light over a cross section of a device. Studies using these techniques have found that the region where the photovoltage is generated in the cell is highly depended on both the doping of the perovskite and the architecture of the cell, specifically on the selective contacts used.^8−11^ A limitation with KPFM and EBIC is that they do not give direct information about the band alignment for buried interfaces within a solar cell structure.

Photoelectron spectroscopy can give information on both the band alignment and photovoltage in perovskite solar cells in addition to the chemical composition and electronic structure. Traditionally, one of the most common methods is ultraviolet photoelectron spectroscopy (UPS), which allows measurement of the position of the Fermi level, valence band maximum (VBM), ionization energy, and work function of a material, and can be extended to provide an estimate of the conduction band minimum (CBM) and electron affinity if the band gap is known. UPS studies have been carried out for a wide range of perovskites,^12,13^ as well as for some selective contacts.^14−17^ Following these measurements, the interfacial band alignment for some systems has been calculated from the electron affinity according to the Andersons rule,^18^ although this method does not always accurately predict the position of the bands.^1^ More lately, PES has been used to measure alignment at buried interfaces. The band alignment is then measured directly for thin film structures with very thin top layers giving the band alignment and bending at the buried interface.^19−22^ Notably, Zu et al. have used this technique to measure the change in band alignment of different interfaces under illumination.^23^ Additionally, by investigating core level spectra, one can determine the chemical properties of the material, allowing the detection of new interfacial chemistry and charge redistributions.^19,20^

However, there are relatively few examples of photoelectron spectroscopy measurements of complete solar cell structures and even fewer during operation. Jaegermann and co-workers have conducted significant work using PES on tapered cross-sectional solar cells to study the chemical profile, band alignment, and internal voltages under different conditions.^24,25^ By measuring a Au/spiro-MeOTAD/perovskite/TiO_2_ cell from the front compact TiO_2_ layer to the Au back electrode, they were able to estimate the position of the VBM, which was compatible with a p–n–n structure, and they detected variations in the Br:I ratio across the cell.^25^ In a followup study, they also showed that the majority of the photovoltage in their systems was generated at the heterojunction between the n-type perovskite and the p-type hole transport material (spiro-MeOTAD).^24^

Another method of studying the interfaces is by increasing the probing depth of the photoelectrons using hard X-ray photoelectron spectroscopy (HAXPES), i.e., by increasing the photon energy and, in turn, the kinetic energy of the photoelectrons.^26^ The larger probing depth allows for studies of buried interfaces below thicker films and, by careful design, for a full solar cell structure to be measured. In this paper, we present a methodology of using HAXPES for direct measurement of the interfacial photovoltage generated between the perovskite, hole transport material (HTM), and electrode in a complete solar cell device during operation. We were able to use the obtained results together with separate measurements on pure materials to determine the band alignment in the heterojunction, both in the dark and at open-circuit conditions under illumination.

## Experimental Methods

### HAXPES Measurements

The measurements were carried out at the GALAXIES beamline at the SOLEIL synchrotron^27^ using a photon energy of 3000 eV, which gives an inelastic mean free path of about 5 nm^28^ and a probing depth (representing 95% of the intensity) of about 15 nm. The synchrotron operated with a ring current of 450 mA, giving an intensity of 3.4 × 10^13^ photons/s at 3000 eV, which was then reduced using a built-in filter to 0.042% of the original intensity. The elliptical X-ray beam has a spot size of about 30 μm (V) x 80 μm (H), giving an area of 1885 μm^2^. With an incidence angle of 2°, this results in an effective spot size of 54,000 μm^2^.^29^ This resulted in an X-ray flux density of 2.62 × 10^13^ photons/s/cm^2^, which corresponds to an irradiance of 1.3 mW/cm^2^. The third-order X-rays, which were not as effectively blocked by the filter, are suppressed by the cutoff energy (6 keV) of the upstream collimating mirror.

The photoelectrons were detected using a Scienta Omicron EW4000 HAXPES hemispherical analyzer, and the measured core levels were fitted using a pseudo-Voigt function^30^ with a polynomial, a Herrera–Gomez,^31^ and a Shirley background.^32^ The intensity (area) of the core levels from the fit was then normalized by photoionization cross section.^33^ The energy scale of the analyzer at both pass energies was validated against the position of the Fermi, Au 4f, and Au 4d levels, shown in Figure S1 and Table S1. The measurements of the cell were performed in fixed mode, i.e., with a constant kinetic energy, using a pass energy of 500 eV, and the measurements of the reference films were performed using swept mode with a pass energy of 200 eV. To remove artifacts introduced by fixed mode, the same region was measured in both fixed and swept modes (Figure S2a). These measurements were then used to calculate a normalization curve (Figure S2b) that was applied to all fixed mode measurements. Each fixed mode core level spectrum was measured, in turn, and then resumed from the first core level with each spectrum saved separately. For the measurement of the selected results, a dwell time of 60, 10, 20 and 5 s was used for Pb 4f, I 3d, C 1s, and Au 4f, respectively, which resulted in a total measurement time of about 150 seconds/iteration with an overhead of about 57%.

The cell was illuminated using a custom-made cold white 100 W LED. It was mounted almost 90° from the surface normal of the sample, relying on the reflectivity of the stainless steel vacuum chamber to evenly distribute the light. The beamline is equipped with a sample holder system for connecting the sample to an external circuit,^34^ allowing the open-circuit voltage and short circuit current generated by the cell to be measured using a multimeter. All measurements were performed at room temperature and at pressures below 10^–9^ mbar.

### Sample Preparation

All perovskite samples were prepared following the method described in our previous work.^29^ In summary, FTO substrates were cleaned using RBS detergent, ethanol, and acetone in an ultrasound bath during several steps of 30 min. Compact TiO_2_ was deposited by spray pyrolysis at 450 °C, obtaining an anatase compact layer of around 20–30 nm of thickness. On top of this layer, 50 μL of a TiO_2_-mp solution was spin-coated at 4000 rpm, with an acceleration of 2000 rpm/s, during 10 s and sintered at 450 °C during 30 min in air, forming a mesoporous scaffold of TiO_2_ nanoparticles. After that, 100 μL of 35 mM lithium bistrifluoromethanesulfonimidate (Li-TFSI) in acetonitrile was spin-coated (3000 rpm for 10 s) and the substrates were thermally annealed again in air at 450 °C for 30 min. After this process, the substrates were brought directly into a glovebox.

Perovskite precursor solutions and thin films were prepared inside a N_2_-filled glovebox. Two master solutions were prepared in advance: (a) 0.9 M PbI_2_ and 0.9 M FAI and (b) 0.9 M PbI_2_ and 0.9 M CsI. The final solution was prepared right before deposition by pouring solution (a) into solution (b) in the proportion a:b = 83:17, obtaining a perovskite final composition of Cs_0.17_FA_0.83_PbI_3_. The solvent was anhydrous DMF:DMSO (4:1) for all solutions. FA salts were bought from Dyesol, lead salts from TCI, solvents from Fisher, and the remaining chemicals from Sigma-Aldrich. All chemicals were used as received without further treatment. Seventy-five microliters of the precursor solution was spread over the substrate and spin-coated using a two-step program. The first step used a rotation speed of 1000 rpm with an acceleration of 200 rpm/s for 10 s, followed by a second step in which the films were spun at 6000 rpm for 15 s using an acceleration of 2000 rpm/s. After 20 ss, 200 μL of anhydrous chlorobenzene was applied on the spinning film. Directly after spin coating, the films were annealed on a hotplate at 100 °C for approximately 1 h.

The polymer P3 was synthesized according to the method described previously.^35^ For deposition, a P3 solution with a concentration of 5 mg/mL in chlorobenzene was prepared and stirred in the ambient environment to fully dissolve the polymer and then spin-coated onto the perovskite or FTO substrates at 3000 rpm for 30 s.

Gold electrodes were evaporated using a Leica EM MED020 thermal evaporator at a pressure below 7 × 10^–3^ mbar. The thickness was measured using a Leica EM QSG100 quartz microbalance. To avoid short circuits, a mask was used to prevent any gold deposition on the side opposite to the etched substrate. A 7 nm gold layer was deposited using this mask; thereafter, the chamber was vented, and an additional mask was added for the evaporation of the thick (>50 nm) gold contact and the bus bars.

## Results and Discussion

### Device Design and HAXPES Characterization

To use this methodology, there are several needs for the design of the solar cell and sample holder:

(1) The solar cell must be functional, meaning that it generates a useful voltage and current
(2) The solar cell and sample holder must allow the application of a bias voltage and measurements of photovoltage and current
(3) The solar cell must allow the detection of core level photoelectrons from the different materials e.g., electrode, HTM, and perovskite, which requires structures with thin films.
(4) The system should be stable under X-rays, voltage bias, and illumination for the duration of the measurements

To fulfill these requirements, suitable materials, deposition methods, and layer thicknesses need to be chosen. Due to the shallow probing depth of photoelectrons, the electrode and the HTM cannot be much thicker than 10 nm to fulfill requirement 3, which is considerably thinner than the optimal layer thicknesses in most perovskite solar cells. This will increase the probability of pin-holes and the likelihood that damage and corrosion of the layers result in device failure, negatively impacting requirement 4.

**Figure 1:**
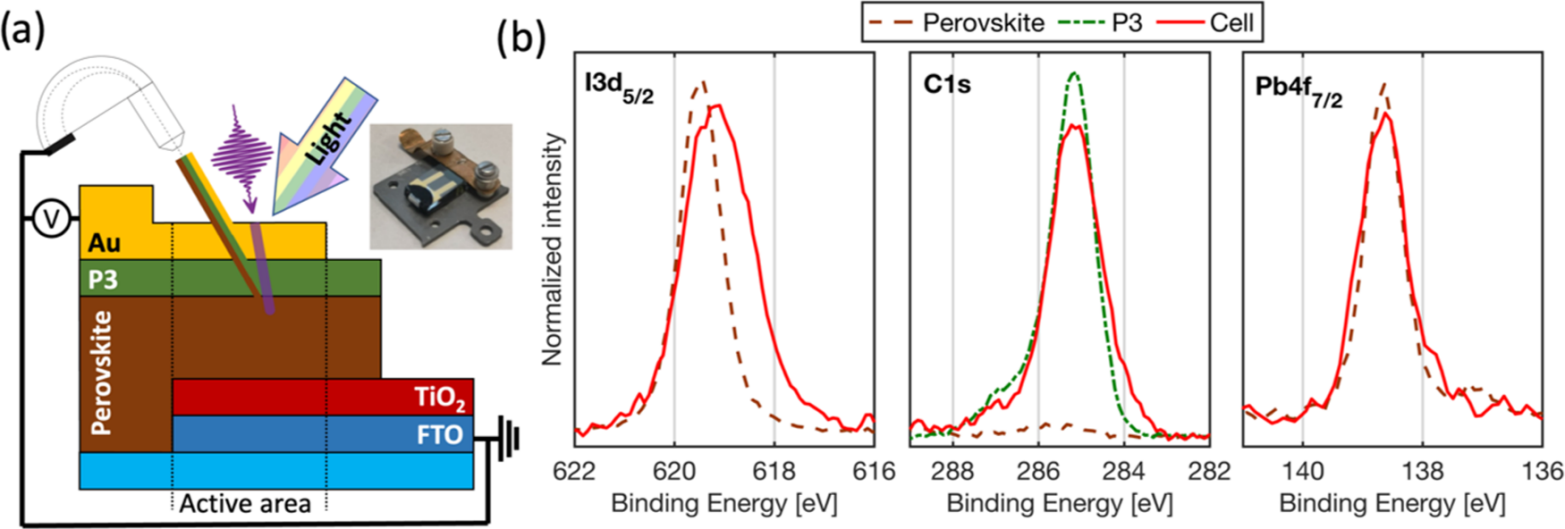
(a) Schematic showing the architecture of the cell, the regions probed by the photoelectrons, and the electrical connections of the solar cell. The inset photograph shows the cell when mounted on the plate and connected to the electrode. (b) The I 3d_5/2_, C 1s, and Pb 4f_7/2_ core levels of the bare perovskite, a P3 reference sample, and of the Cs_0.17_FA_0.83_PbI_3_ /P3/Au interface in the solar cell device. The signals from the uncovered bare perovskite were intensity-normalized and energy-calibrated to Pb 4f_7/2_ at 138.7 eV, while the signals from P3 were normalized against the total C 1s intensity and energy-calibrated against the most intense C 1s signal.

A possible cell architecture is shown in Figure 1a. The FTO and TiO_2_ were removed from the substrate in the areas below the thick gold contact. This removal reduces the risk of short-circuiting the device upon connection to external contacts. This region also does not generate any current under illumination by light or X-ray, allowing us to find it by moving the X-ray spot over the sample. Gold was chosen as a back electrode due to its corrosion resistance, high conductivity, and ease of depositions by evaporation. A gold average thickness of about 7 nm was chosen, as the resistivity increased substantially if it was made any thinner.^36,37^ At this thickness, the evaporated gold does not form a continuous film but interconnected islands, allowing more of the photoelectrons to escape to the detector.^38^ This thin back electrode, although conductive, resulted in a high series cell resistance. The cell design therefore included bus bars of higher gold thickness, reducing the series resistance significantly and improving the reliability of the cells.

If the gold was deposited directly on the perovskite without any selective contact, the perovskite would degrade rapidly by forming Pb^0^ under X-ray illumination even at intensities where the perovskite alone was stable, which we attributed to increased beam damage from the generation of additional secondary electrons in the gold and reactions catalyzed by gold like those reported by Kerner et al.^39^ The cells were therefore fabricated with a HTM layer acting as a buffer between the metal electrode and perovskite, which increases the stability and efficiency of the cells and mirrors what is used in most perovskite solar cell designs. In the selected results shown below, a 10 nm polymeric HTM, referred to as P3 (structure shown in Figure S4a),^35^ was deposited by spin coating. This polymer gives efficiencies comparable to those of spiro-MeOTAD but does not require any dopants or additives. Furthermore, the optimal thickness of the P3 layer is about 30 nm, significantly thinner than for spiro-MeOTAD,^40^ and the surface roughness is low (below 5 nm), reducing the risk of shunting.^35^ The perovskite composition used in the experiments was Cs_0.17_FA_0.83_PbI_3_, which cannot exhibit anion phase segregation^41^ and is resistant to the formation of metallic lead as shown in our previous studies.^29,42^

Figure 1b shows the I 3d_5/2_, C 1s, and Pb 4f_7/2_ core levels from the perovskite reference film, the P3 reference film (i.e., the hole conductor), and from the Cs_0.17_FA_0.83_PbI_3_/P3/Au interface of the solar cell. The full characterization of the reference films is shown in Figures S3 and 4b and Table S2. The Pb 4f_7/2_ core level shows only one contribution at 138.6 eV binding energy for both the interface and the perovskite reference, which originates from Pb^2+^ in the perovskite. The C 1s core level shows one intense peak at approximately 285.1 eV for the cell that is not present in the perovskite reference. This peak can therefore be assigned to carbon in the P3 polymer. The core levels are slightly wider for the thin film solar cell measurements than for the references, which could be due to the use of a higher pass energy (see below), which gives an energy resolution of about 0.64 eV (Table S1). Taken together, these core level results are consistent with the general structure in Figure 1a.

However, for the I 3d_5/2_ core level, there are considerable differences between the uncovered perovskite layer and when incorporated into the device structure. The I 3d_5/2_ is significantly broader in the measurements on the device structure compared to that on the uncovered perovskite layer, showing additional intensity at lower binding energies. This suggests that an additional iodide compound is present in the device structure observed at lower binding energy. The total I/Pb ratio is 4.9 in the device structure, which is higher than the value of 3.8 observed for the uncovered perovskite layer. This difference could arise due to the diffusion of iodide into the organic HTM layer, which has been widely reported.^43,44^ Lead, on the other hand, is significantly less mobile and has, to the best of our knowledge, not been observed to diffuse into the HTM layers. Therefore, in the choice between I 3d or Pb 4f as a representative signal from the perovskite layer, it is better to use the Pb 4f core level.

The inset in Figure 1a shows the cell mounted to the sample plate designed for external voltage bias measurements at the GALAXIES beamline. In this configuration, the TiO_2_/FTO substrate is grounded, while the gold electrode is connected to an external circuit allowing voltage to be measured or applied across the cell during PES measurements. The illumination setup was designed to ensure that the sample was sufficiently illuminated to create charge separation but not to simulate the solar spectrum. During illumination in the measurement chamber, the cell generated a maximum short circuit current of about 0.1 mA with an estimated active area of about 0.16 cm^2^, resulting in a current density of 0.63 mA/cm^2^. The X-ray irradiance was reduced to about 1.3 mW/cm^2^, and the setup was configured to allow efficient detection at the expense of energy resolution to minimize X-ray-induced beam damage.

### Operando HAXPES Results

**Figure 2:**
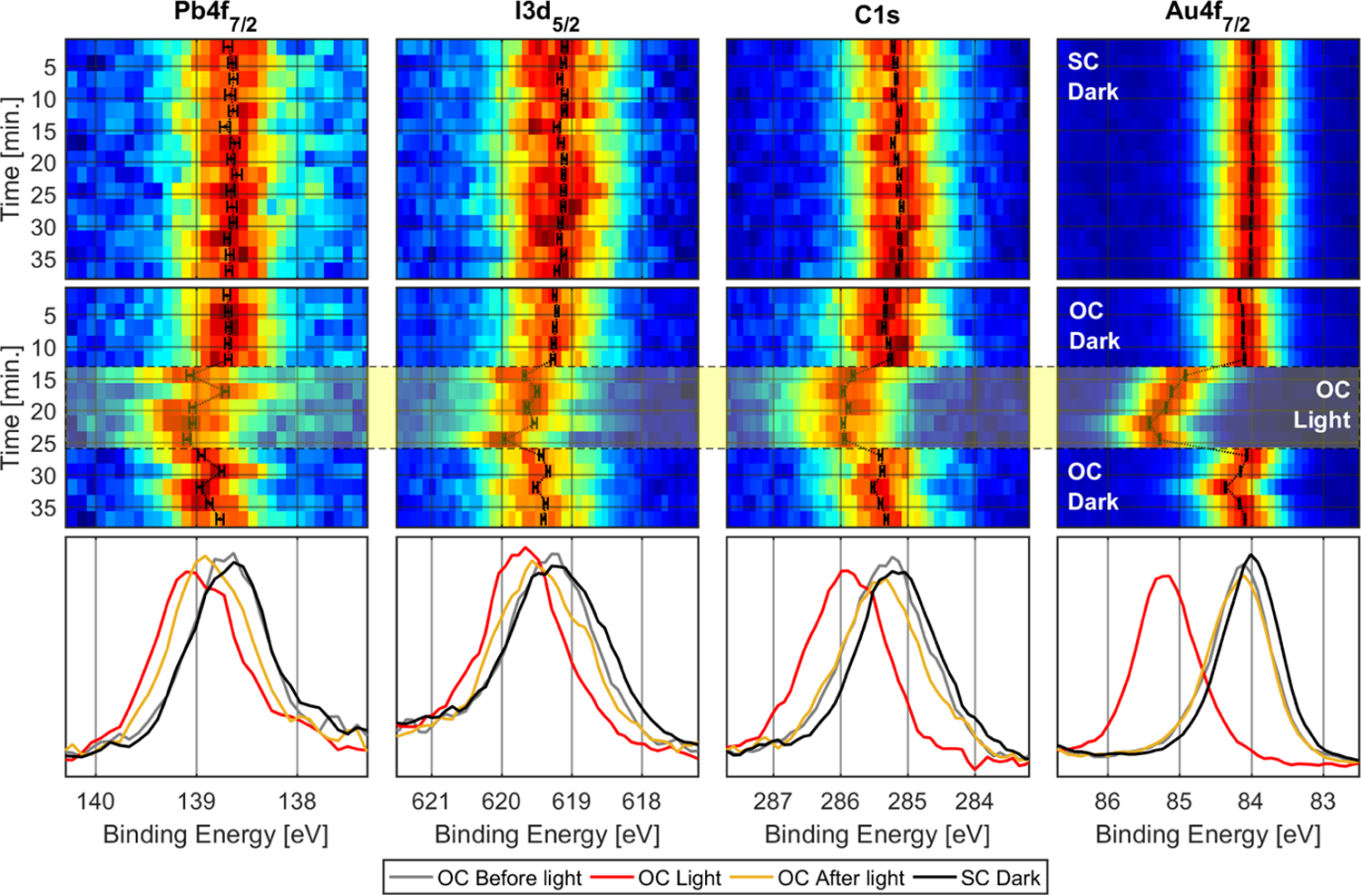
(Top row) Map of intensity vs. binding energy and time of the Cs_0.17_FA_0.83_PbI_3_/P3/Au interface in the solar cell device during short circuit in the dark. The error bars indicate 95% confidence interval of the binding energy of each core level (determined from the Voigt fit). (Middle row) The corresponding map during open circuit with and without light. (Bottom row) The average of the core level spectra recorded during short circuit in the dark and open circuit before, during, and after illumination. Binding energy calibrated against Au4f during SC in the dark before illumination at 84 eV.

Figure 2 shows the time evolution of the Pb 4f_7/2_, I 3d_5/2_, C 1s, and Au 4f_7/2_ core levels of the Cs_0.17_FA_0.83_PbI_3_/P3/Au interface in the solar cell device. The measurements were recorded with the cell at short circuit in the dark (Figure 2, top row) and at open circuit with a period of illumination (Figure 2, middle row). The bottom row of Figure 2 shows the average spectra at each condition. All core levels were fitted using a Voigt function to determine the core level binding energy, of which the 95% confidence interval for the peak position is indicated by the black error bars. When the cell is short-circuited, the binding energy of all core levels remained relatively constant. However, when the cell is at open circuit during illumination, we observe shifts in the core levels toward higher binding energies compared to the short circuit conditions. We attribute this to the generation of a photovoltage, splitting of the Fermi level between the grounded front contact (FTO), and the gold electrode at the back contact. In essence, the back of the solar cell becomes positively charged by the generated photovoltage, making it more difficult for the photoelectrons to escape. We were also able to measure the changes in the Pb/Au, I/Au, C/Au, and I/Pb intensity ratios over time (Figure S5). There are some minor changes in the ratios; however, they were close to that of the uncertainty of the fit.

**Figure 3:**
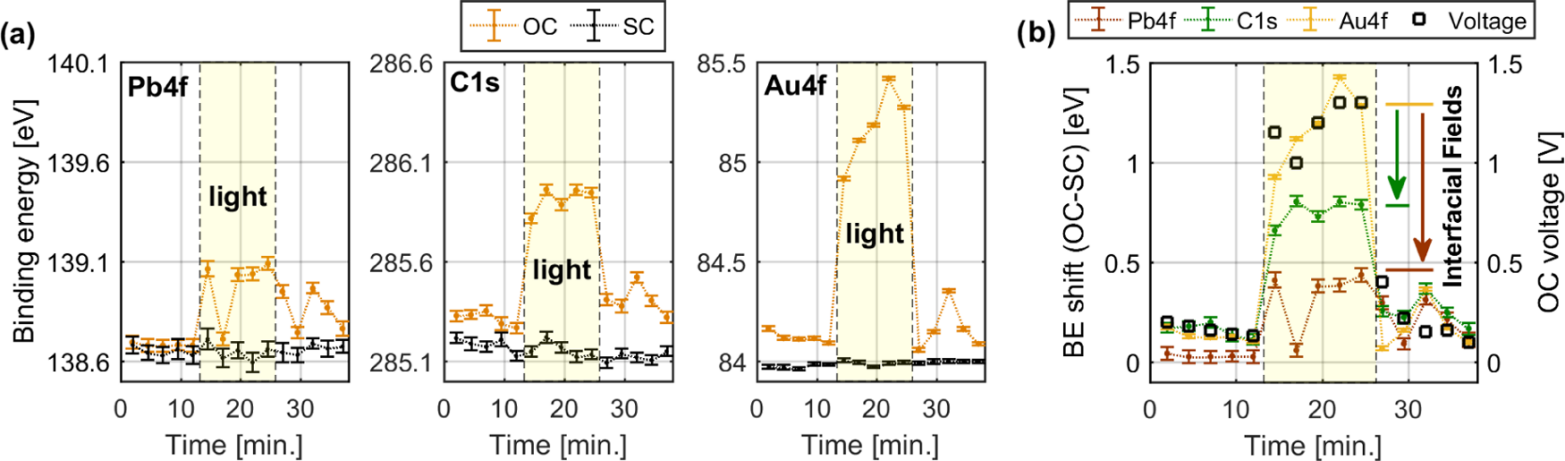
(a) Binding energy as a function of time of the Pb 4f_7/2_, I 3d_5/2_, C 1s, and Au 4f_7/2_ of the Cs_0.17_FA_0.83_PbI_3_/P3/Au interface in the solar cell device at short circuit in the dark and under open circuit with and without light. Calibrated against Au 4f_7/2_ during short circuit in the dark at 84 eV. (b) The binding energy shift in open circuit in relation to the average binding energy in short circuit.

Figure 3a shows the binding energy of the Pb 4f_7/2_, C 1s, and Au 4f_7/2_ core levels over time. When the cell is measured in the dark, there are no significant shifts with time, meaning that the interface is stable under X-ray irradiation for the duration of the measurement. A small shift toward higher binding energies of the core levels (most clearly visible in the Au 4f signal) is observed in open circuit relative to short circuit, which is due to photovoltage generated by the cell from the X-ray beam. During illumination, the binding energy shifts relative to the short circuit condition become significantly larger as the photovoltage generated by the cell increases. When the light is removed, most of the shift disappears, although the core levels are at slightly higher binding energies compared to before illumination. Such shifts indicate that the illumination with visible light does not only gives rise to the electron–hole pair separation, but that the illumination also may induce some irreversible chemistry, such as the loss of iodine.

Figure 3b shows the binding energy shift of the Pb 4f, C 1s, and Au 4f core levels when the core levels are measured at open circuit relative to when the cell is at closed circuit in the dark. The photovoltage generated by illuminating the cell was also measured externally using a voltmeter, and the numbers have been overlaid in Figure 3b as a comparison. The shift in the Au 4f signal correlates well with the voltage generated by the cell, although there are some discrepancies partly due to the voltage being recorded at the beginning and Au 4f being measured at the end of each iteration. This shows that the shift in the Au 4f core level is due to the photovoltage generated by the cell. Similarly, this would also suggest that the shifts observed in the C 1s and Pb 4f core levels correspond to the internal photovoltage generated in the device stack up to the HTM and perovskite layer, respectively. However, the magnitude and behavior of the shift vary depending on the core level, with an average shift of 1.19 ± 0.19, 0.76 ± 0.07, and 0.33 ± 0.16 eV for the Au 4f, C 1s, and Pb 4f core levels, respectively.

The shifts that we observe give information about the semiconductor device physics. Photoelectrons from core levels are affected when passing internal electric fields and therefore will respond to changes in those fields, i.e., the core level positions are sensitive to changes in internal electric fields. When light is applied to our solar cell, electrons and holes will be excited to the conduction and valence band resulting in a built-up of the quasi-Fermi levels for electrons and holes within the perovskite. The holes will be extracted over the interface from the perovskite to the HTM and then to the gold electrode. As a result, the Fermi level of the gold is then expected to adjust with the quasi-Fermi level of the holes in the perovskite. As the cell is operating at open circuit, these holes will not be extracted into the external circuit, and they will instead generate a photovoltage by this light-driven redistribution of charges. In a system with mobile ions, as is the case for the perovskite solar cell investigated here, the charge redistribution may include both movement of ions and electrons/holes. In the end, these interactions will result in a steady state, in which the charge separation currents generated by the excitation are balanced by opposite recombination currents. In this photo-induced steady state, the internal electric fields are redistributed compared to the equilibrium state in the dark. By following the changes in the core levels for the different materials, we can detect absolute changes in potential between the different layers in our experiment. From these, we can determine how the change in the internal electric field is distributed among the different materials.

The total electric field change across the device corresponds to the change in the Au 4f core level position of 1.19 eV and reflects the open-circuit voltage of about 1.2 V. The electric field change from Au up to the P3 layer can be obtained from the difference between the shift in Au 4f and C 1s, i.e., 1.19 eV compared to 0.76 eV. This difference of 0.43 eV thus corresponds to an internal photovoltage of 0.43 V. Similarly, the photovoltage between the P3 layer and the perovskite, reflected by the binding energy shifts of 0.77 and 0.33 eV, is determined to be 0.43 V. From this, we can conclude that roughly 70% of the open-circuit voltage (0.86 of 1.2 V) is generated at the back interfaces, evenly distributed between the perovskite/HTM (0.43 V) and HTM/Au interfaces (0.43 V). These internal voltages could stem from changes in the interfacial dipole or from charge redistributions extending over the measured region below the interface. For the HTM, this region could extend over the whole layer (the layer is thinner than the probing depth of 15 nm of the measurement). For the perovskite, this probing depth means that the average Pb 4f photoelectron comes from about 5 nm (roughly 8 unit cells) below the perovskite/P3 interface.

Electric field changes might also occur at the perovskite/TiO_2_ and TiO_2_/FTO interfaces due to electron accumulation, but these cannot be detected in our experiment, as we are only able to measure the perovskite/HTM/gold interface due to the probing depth of our experiment.

To the best of our knowledge, the application of this methodology to investigating buried interfaces in functional perovskite solar cell structures is rather new, but there are complementary techniques with which we can compare our finding. A study by Wussler et al. using PES on tapered cross-sectional Au/spiro-MeOTAD/perovskite/TiO_2_ cells found that the majority of the photovoltage was generated at the interface with, or even within, the spiro-MeOTAD layer, which supports our results.^24^ Some Kelvin probe force microscopy studies show similarities with our results, even if the interface at which the photovoltage was generated was demonstrated to depend on the architecture and the doping of the perovskite. Cai et al. showed that the photovoltage was generated at the HTM/perovskite interface for an n-type perovskite but at the electron transport layer (ETL)/perovskite interface for a p-type perovskite.^8^ Hermes et al. found that the photovoltage could be generated at the interface of both the HTM and the ETL with the perovskite. However, depending on the cell architecture, the region where the photovoltage was generated could extend deep into the HTM all the way to the interface with the gold electrode, similar to what is observed in the present study.^9^

### Energy Level Alignment

**Figure 4:**
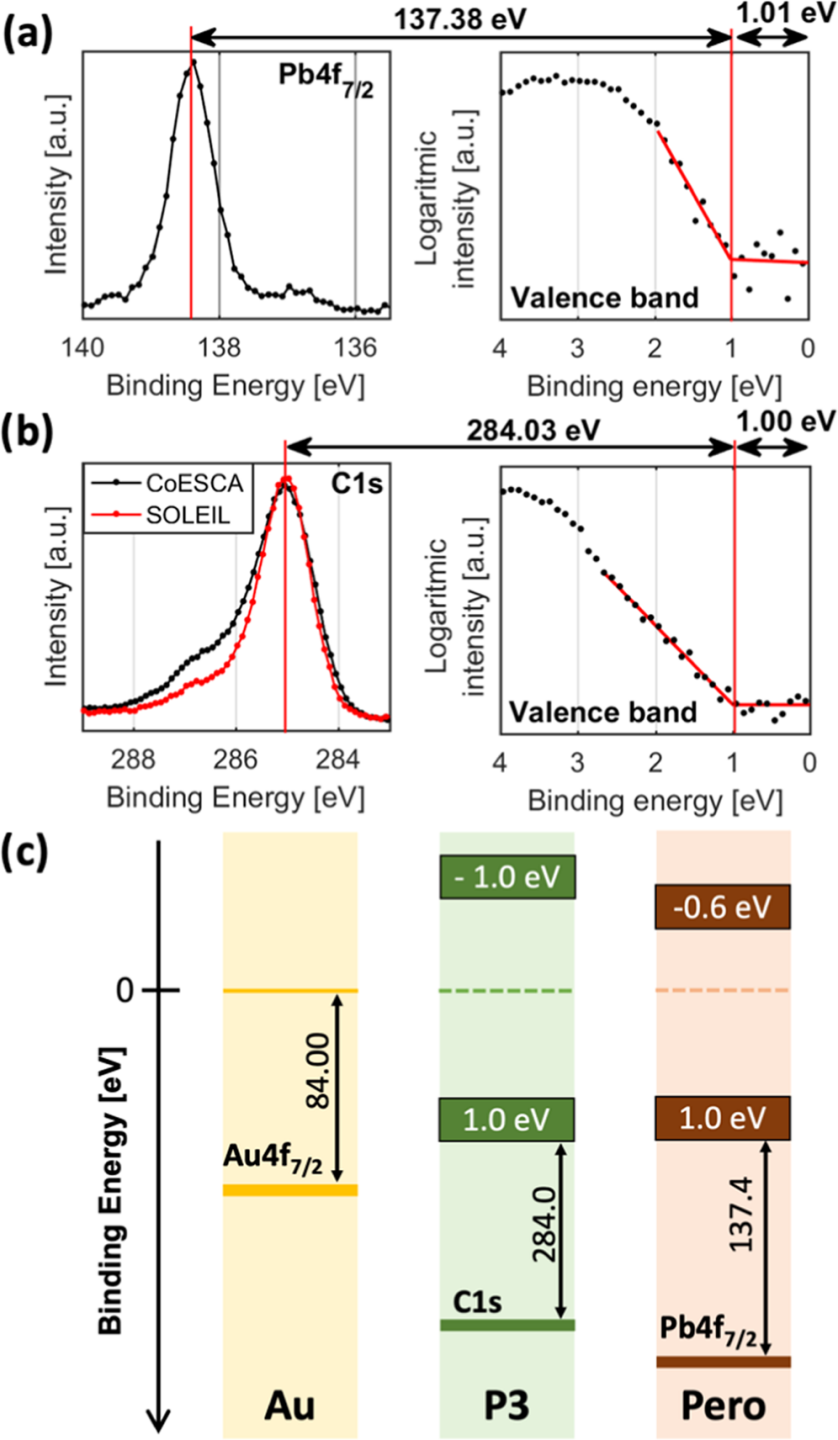
(a) Pb 4f_7/2_ and valence band edge of the perovskite sample, measured at SOLEIL using a photon energy of 3000 eV. (b) The C 1s and valence band edge of the P3 sample measured at CoESCA using a photon energy of 535 eV (in black) and measured at SOLEIL using a photon energy at 3000 eV (in red). For calibration, see the Supporting Information. (c) The position of the Fermi level at 0 eV binding energy, valence band maximum (VBM), and conduction band minimum (CBM) together with the core level to Fermi/VBM binding energy difference of the gold electrode, P3, and perovskite.

The core level measurements described above can also be used to estimate the band alignment at the Cs_0.17_FA_0.83_PbI_3_/P3/Au interface in the dark and under illumination. For this purpose, reference measurements of the valence band of pure P3 and perovskite samples are used. Figure 4a shows the Pb 4f_7/2_ and valence band edge of a bare Cs_0.17_FA_0.83_PbI_3_ perovskite thin film, energy-calibrated against a separately measured gold sample. All core levels were fitted with a Voigt profile, while the VBM was determined using a logarithmic extrapolation, which has been shown to give more realistic values for the position of the VBM.^45−47^ For comparison with other studies, the values from the fits will be given with two decimals even if the accuracy is likely not better than 0.1 eV. The measurements place the VBM at 1.01 eV below the Fermi level. Given a band gap of about 1.6 eV, this means that the CBM is 0.6 eV above the Fermi level and that the perovskite is n-type. Combining the measurement of the valence band and the core levels, we can determine the Pb 4f_7/2_ and VBM binding energy difference to be 137.38 eV. This should be a material constant for a specific perovskite composition and be unaffected by the position of the Fermi level and doping. The main uncertainty in this procedure comes from the extrapolation for determining the VBM. The value was therefore validated by measuring an identical thin film at the I09 beamline at DIAMOND, equipped with an identical spectrometer, using the same photon energy (Figure S6 and Table S3). The Pb 4f_7/2_ to the VBM binding energy difference was found to be 137.46 eV. This is slightly larger than 137.38 eV, which most likely is due to inaccuracies in the determination of the VBM. For the remainder of this study, we will use a Pb 4f_7/2_ to the VBM difference of 137.4 eV. Compared to the literature, we find that Hellmann et al. determined the Pb 4f_7/2_ to VBM to be 137.19 eV for MAPbI_3_, while Wussler et al. determined it to be 137.39 eV for FA_0.85_MA_0.15_Pb(I_0.85_Br_0.15_)_3_.^24,48^

Measuring the valence band of the organic hole transport layer accurately at a photon energy of 3000 eV is difficult given the low photoionization cross section of C 2p orbitals, which dominate the valence band. However, using a lower photon energy of 535 eV results in a C2p cross section that is 600 times greater than at 3000 eV.^33,49^ We therefore carried out additional measurements of the C 1s, S 2p, and valence band at the CoESCA endstation at BESSY-II using a photon energy of 535 eV (see the Supporting Information for details). From this, we are able to determine that the VBM of the P3 is 1.00 eV below the Fermi level (Figure S7 and Table S4), i.e., very similar to the Cs_0.17_FA_0.83_PbI_3_. With a band gap of about 2.0 eV, this places the Fermi level in the middle of the band gap, which indicates that P3 is an intrinsic semiconductor. We are also able to determine the binding energy difference between the most intense C 1s signal and the VBM to be 284.03 eV (Figure 4b), rounded to 284.0 eV. A diagram of the binding energy of the VBM and CBM relative to the Fermi level and of the core level to the VBM binding energy difference of all samples is shown in Figure 4c.

Using the core level to VBM binding energy differences, we can estimate the binding energy of the VBM from the binding energy of the core levels, even when the valence band cannot be measured directly. This can be used to determine the band alignment in buried interfaces by just measuring the core levels, a technique summarized by Kraut et al.^50^ The measured position of the band represents not only the position directly at the interface but instead the interface region within the exponentially weighted probing depth. For the perovskite, the probing depth is about 15 nm, with the average Pb 4f photoelectron coming from about 5 nm below the perovskite/P3 interface.

**Figure 5:**
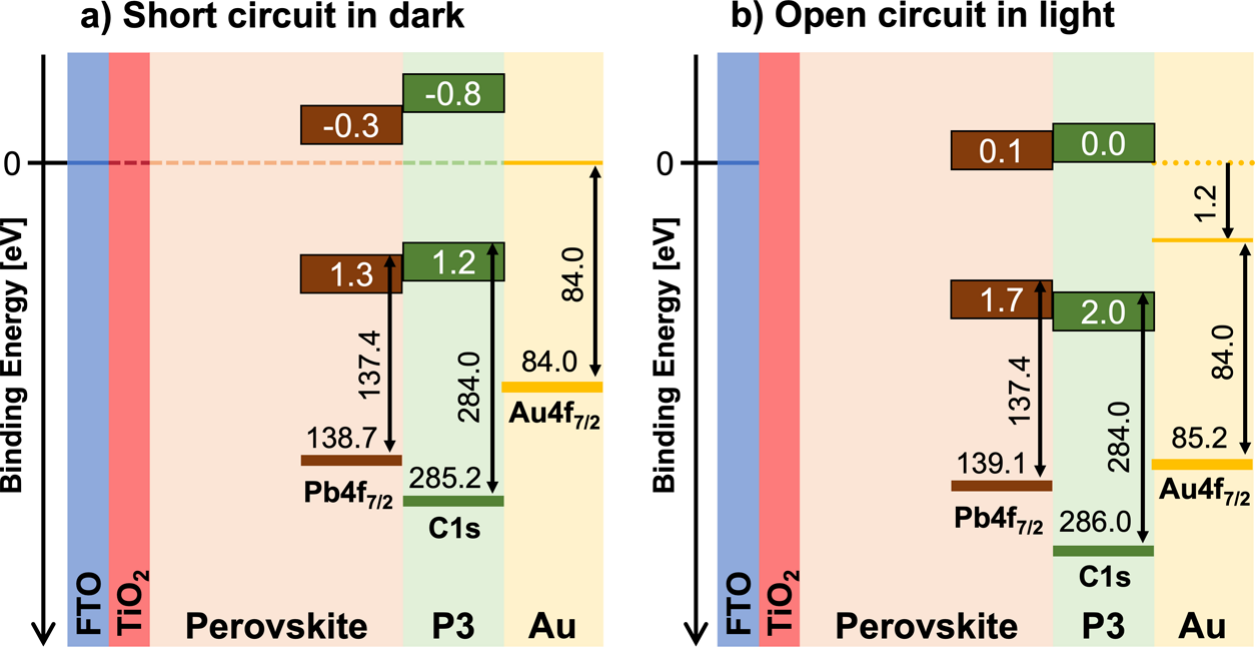
Valence band alignment as determined from the core level to VBM binding energy difference and band gap for the solar cells in short circuit in the dark (a) and open circuit during illumination when generating a voltage of 1.2 V (b). The relative positions of core levels and valence and conduction bands are kept constant in the figure, which would be expected for a system where the charge redistribution is concentrated at the interfaces leaving the bulk material of each layer intact. The dashed line in panel (a) indicates the Fermi level at 0 eV binding energy since the FTO is grounded to the spectrometer. In panel (b), the Fermi level of FTO is at 0 eV binding energy since the FTO is still grounded and the Fermi level of Au is at 1.2 eV binding energy representing the 1.2 V open-circuit voltage. The binding energy axis is not to scale.

When the cell is short-circuited in the dark, the binding energies of the Pb 4f_7/2_, C 1s, and Au 4f_7/2_ core levels are 138.7, 285.2, and 84.0 eV. This positions the VBM of the perovskite and P3 at 1.3 and 1.2 eV, respectively, with the CBM at −0.3 and −0.8 eV, respectively. The corresponding band diagram is shown in Figure 5a and represents the energy alignment at the back contacts of the solar cell at equilibrium. However, during illumination, when the cell is generating 1.2 V, the binding energy of the Au 4f core level and thus the Fermi level of the back contact will shift by almost 1.20 eV compared to when measured in the dark at equilibrium. As also indicated in the discussion above, the corresponding shifts of Pb 4f and C 1s are smaller, and we measure shifts of about 0.4 and 0.8 eV, respectively. We therefore expect the VBM binding energy of the perovskite and P3 to be 1.7 and 2.0 eV, respectively, with the CBM at 0.1 and 0.0 eV, respectively, relative to the Fermi level of the FTO and the spectrometer. A band diagram based on these values is shown in Figure 5b and represents the energy alignment at the back contacts of the solar cell at open circuit during illumination. The band diagrams shown in Figure 5 are based on the core level positions observed. For the case of a thin interfacial dipole layer, these positions represent the energy levels throughout the material. However, for the case of extended electrical fields (i.e., band bending within the measured layers), the positions correspond to average band positions at the back contact of the solar cell in the two different cases (short circuit dark and open-circuit light). Thus, the measurements do not resolve any built-in electric fields (band bending), which could be present at these interfaces and change between the two cases.

If we compare the positions of the bands at short circuit in the dark to when measuring the pristine materials (Figure 4c), we find that the average VBM of the P3 has shifted to higher binding energies by about 0.2 eV and the average VBM of the perovskite by about 0.3 eV, resulting in an aligned VBM with a small offset that allows holes to be extracted. At the same time, the conduction band alignment based on the band gaps confirms that there is about a 0.5 eV barrier to extraction of electrons. When the cell is illuminated, the valence bands are no longer well aligned and there appears to be a 0.3 eV barrier to the extraction of holes. At the same time, the barrier to hole extraction in the conduction band has decreased to 0.1 eV, making unwanted extraction of electrons into the HTM more likely. While this is not the first time these effects are observed (similar effects have been observed by Zu et al.^23^), to the best of our knowledge, the present investigation is the first time the effects have been followed in a functioning solar cell structure. These observations have implications for the material design of solar cell interfaces. Commonly, materials are chosen by the alignment of valence and conduction bands with the assumption that these will remain more or less identical in the assembled device. However, our results indicate significant band realignment of the bands at interfaces in the device during illumination at open-circuit conditions.

## Conclusions

To conclude, we have been able to design a functioning solar cell device that allows for simultaneous HAXPES measurement of the layers (Au, HTM, and perovskite) at the back contact and thus the voltage generation. While operating this cell at open circuit, both with and without illumination, we were able to observe the open-circuit voltage generated by the cell through the changes in core level positions. A different magnitude of shift was observed for the different layers at the back contact with the largest shift present in the gold. These results suggest that a significant part of the photovoltage is generated at the interface to the HTM and gold contact. Using complementary valence band measurements, we were able to estimate the band alignment of the interfaces both during equilibrium and at open circuit during illumination from this data and show that the band alignment changes during illumination. These changes in band alignment could result from chemical changes at the interface during illumination or from charge redistributions, i.e., a difference in how the electric field across the device is redistributed upon illumination. This includes the diffusion of mobile ions across interfaces, which was found to have occurred prior to the operando measurements with the diffusion of iodide into the HTM. Overall, we therefore demonstrate that this method can be useful for investigating voltage generation, band alignment, and chemical changes of perovskite and other types of solar cell under working conditions. However, for this method to reach its full potential, further development in the sample design is required to both increase stability and decrease the series resistance of the gold electrode.

## Supporting Information Available

Spectrometer characterization and fixed mode normalization; and PES characterization of Cs_0.17_FA_0.83_PbI_3_ and P3 reference films and core level to VBM binding energy differences (PDF)
