# Supplementary material for: Direct Measurements of Interfacial Photovoltage and Band Alignment in Perovskite Solar Cells Using Hard X-ray Photoelectron Spectroscopy
Source: ACS Appl Mater Interfaces. 2023 Feb 27;15(9):12485–94. doi: 10.1021/acsami.2c17527 (PMC9999345; doi:10.1021/acsami.2c17527)
Supplement: Supplementary file 1 — am2c17527_si_001.pdf [file am2c17527_si_001.pdf]

Supporting information:

Direct measurements of interfacial photovoltage and band alignment in perovskite solar cells using hard X-ray photoelectron spectroscopy

Sebastian Svanström,[1] Alberto García-Fernández,[2] Tamara Sloboda,[2] T. Jesper Jacobsson,[3] Fuguo Zhang,[4] Fredrik O. L. Johansson,[5,6] Danilo Kühn,[5] Denis Céolin,[7] Jean-Pascal Rueff,[7,8] Licheng Sun,[4,9,10] Kerttu Aitola,[11] Håkan Rensmo,[1,*] Ute B. Cappel[2,*]

1: Condensed Matter Physics of Energy Materials, Division of X-ray Photon Science, Department of Physics and Astronomy, Uppsala University, Box 516, SE-751 20, Uppsala, Sweden

2: Division of Applied Physical Chemistry, Department of Chemistry, KTH - Royal Institute of Technology, SE-100 44 Stockholm, Sweden

3: Institute of Photoelectronic Thin Film Devices and Technology, Key Laboratory of Photoelectronic Thin Film Devices and Technology of Tianjin, College of Electronic Information and Optical Engineering, Nankai University, Tianjin 300350, China

4: Division of Organic Chemistry, Department of Chemistry, KTH - Royal Institute of Technology, Stockholm SE-100 44, Sweden

5: Institute for Methods and Instrumentation in Synchrotron Radiation Research FG-ISRR, Helmholtz-Zentrum Berlin für Materialien und Energie Albert-Einstein-Strasse 15, 12489 Berlin, Germany

6: Institut für Physik und Astronomie, Universität Potsdam, Karl-Liebknecht-Strasse 24-25, 14476 Potsdam, Germany

7: Synchrotron SOLEIL, L'Orme des Merisiers, BP 48 St Aubin, 91192 Gif sur Yvette, France

8: Laboratoire de Chimie Physique-Matière et Rayonnement, Sorbonne Université, CNRS, 75005 Paris, France

9: State Key Laboratory of Fine Chemicals, Institute of Artificial Photosynthesis, DUT−KTH Joint Education and Research Centre on Molecular Devices, Dalian University of Technology (DUT), Dalian 116024, China

10: Center of Artificial Photosynthesis for Solar Fuels, School of Science, Westlake University, Hangzhou 310024, China

11: New Energy Technologies Group, Department of Applied Physics, Aalto University School of Science, Box 15100, 00076 AALTO, Finland.

*hakan.rensmo@physics.uu.se

*cappel@kth.se

Spectrometer energy scale calibration and resolution

The energy scale of the spectrometers was validated using the fit of the Fermi edge, and the Au $4f_{7/2}$ and the Au $4d_{5/2}$ core levels, shown in Figure S1. Comparing to reference values (Table S1), the binding energy scale deviates with less than 0.12 % from the reference values and, more importantly, this deviation is consistent between the two different pass energies. We also find that the energy resolution at a pass energy of 500 eV is about double that at 200 eV.

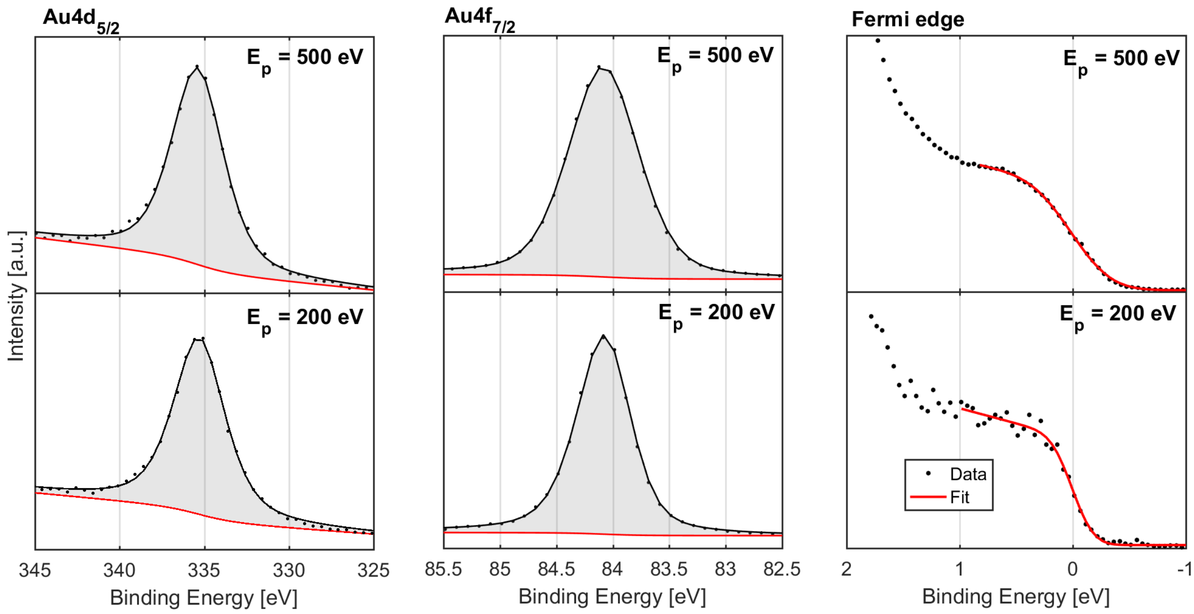

Figure S1: The fit of the Au $4d_{5/2}$, Au $4f_{7/2}$ core level and Fermi edge of gold sample measured at two different pass energies. Measured at the GALAXIES beamline at the SOLEIL synchrotron using a photon energy of 3000 eV.

Table S1: The reference binding energies and measured binding energies (and energy resolution) of the Fermi edge, Au$4f_{7/2}$ and Au $4d_{5/2}$ core level at a pass energy of 200 eV and 500 eV.

	Reference [1]	SOLEIL (E_p = 200 eV)	SOLEIL (E_p = 500 eV)
Energy resolution [eV]	-	0.32	0.64
Fermi [eV]	0.00	0.00	0.00
Au $4f_{7/2}$ [eV]	83.98	84.08	84.08
Au $4d_{5/2}$ [eV]	335.22	335.22	335.33
Error Au $4f_{7/2}$ - Fermi	-	+ 0.12 %	+ 0.12 %
Error Au $4d_{5/2}$ - Au $4f_{7/2}$	-	- 0.04 %	+ 0.03 %

Fixed mode normalization

In fixed mode, unlike the more common swept mode, the selected kinetic energy of the analyser is kept constant which increases the effective collection time. However, it also introduces artefacts due to the shape of the MCP and variations in the sensitivity of its pixels which are not present in swept mode. To compensate for these artefacts a normalization curve can be calculated by measuring a region using both swept mode and fixed mode with the same step size (Figure S2a). This normalization curve can then be used to reduce the effect of the artefacts, shown in Figure S2b. We have found that the shape of the normalization curve is independent of the kinetic energy of the electron but is affected by lens mode and pass energy.

The fixed mode core level measurements were carried out in cyclic mode, each core level spectrum measured in turn and then restarted from the first core level, with each cycle saved separately. The detector had a dwell time of 1 s, i.e. a readout rate of 1 Hz, with the number of readouts (iterations) adjusted depending on the intensity of the core level.

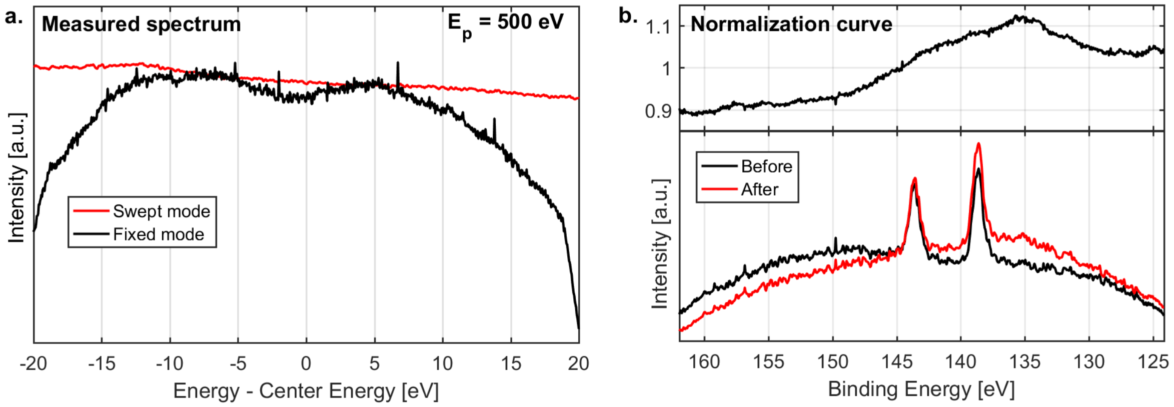

Figure S2: a) A spectrum of the same, featureless, region in swept mode and fixed mode with a pass energy of 500 eV. b) The effect on fixed mode normalization on the shape of the curve (normalized to average intensity).

Reference sample characterization

Figure S3 show the Cs 4d, I 4d, Pb 4d, I 3d, Pb 4f, N 1s and C 1s core levels and the valence band of the $Cs_{0.17}FA_{0.83}PbI_3$ reference films measured at the GALAXIES beamline at SOLEIL and I09 beamline at DIAMOND. The binding energies of all core levels at DIAMOND are slightly higher than at SOLEIL. In addition, there is slightly more I^- and Cs^+ in relation to Pb^{2+} in the sample measured at DIAMOND. We also observe some formation of metallic lead at SOLEIL but not at DIAMOND, presumably because of the lower X-ray flux densities at the latter.[2] Table S2 shows the binding energy, width (FWHM) of the core levels shown in Figure S3 as well as the elemental ratio relative to Pb^{2+} derived from the area of the photoelectron peak normalized to the photoionization cross section.

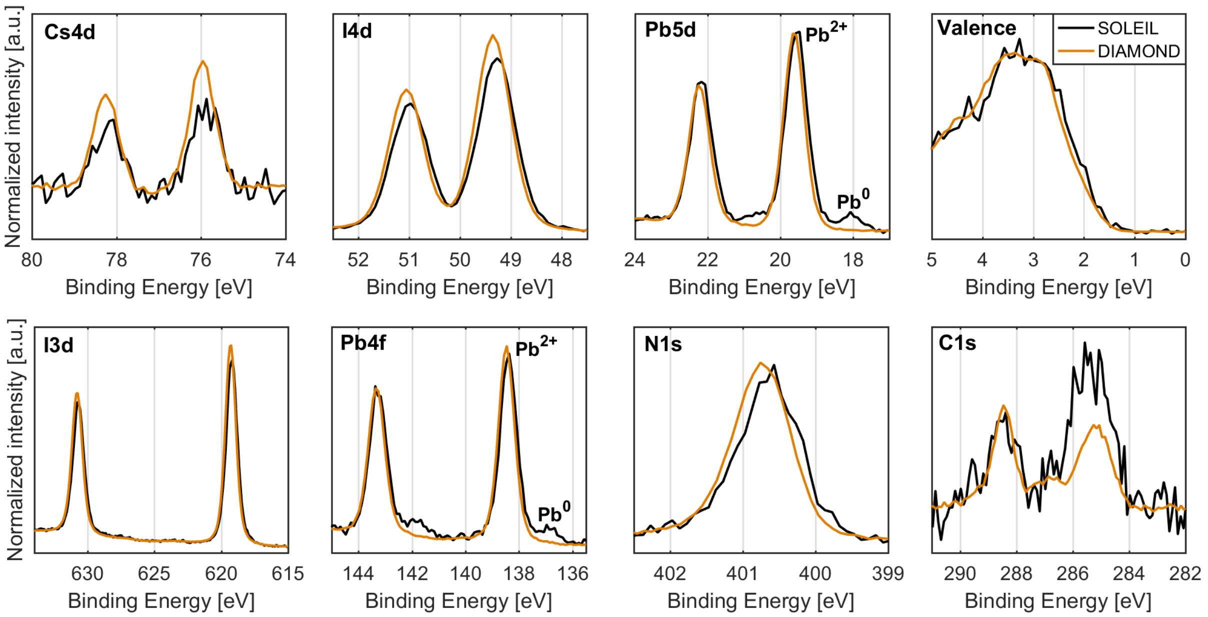

Figure S3: The valence band and core level spectra of the $Cs_{0.17}FA_{0.83}PbI_3$ reference sample, measured at the GALAXIES beamline at SOLEIL and at the I09 beamline at DIAMOND referenced against Au $4f_{7/2}$ at 84.0 eV. Measured at a photon energy of 3000 eV and intensity normalized to the Pb^{2+} signal of the Pb 4f core level.

Table S2: The binding energy in eV of the main doublet and width (FWHM) in eV of the core levels and elemental ratios of the $Cs_{0.17}FA_{0.83}PbI_3$ reference sample measured at SOLEIL and DIAMOND.

**Binding energy**	**VBM**	**Pb 5d**	**I 4d**	**Cs 4d**	**Pb 4f**	**N 1s**	**I 3d**
SOLEIL	1.01	19.56	49.27	75.88	138.39	400.63	619.21
DIAMOND	1.01	19.62	49.35	75.97	138.46	400.74	619.30
**Width (FWHM)**	**-**	**Pb5d**	**I4d**	**Cs4d**	**Pb4f**	**N1s**	**I3d**
SOLEIL	-	0.70	0.86	0.72	0.75	0.98	0.97
DIAMOND	-	0.67	0.81	0.72	0.72	0.93	0.94
**Ratios**	**-**	**Pb 5d/Pb 4f**	**I 4d/Pb 4f**	**Cs 4d/Pb 4f**	**-**	**N 1s/Pb 4f**	**I 3d/Pb 4f**
SOLEIL	-	1.03	3.28	0.13	-	1.62	3.84
DIAMOND	-	0.94	3.46	0.20	-	1.67	3.85

Figure S4a shows the monomer structure of P3, while Figure S4b shows the S 1s, F 1s, C 1s and S 2p core levels of the bare P3 polymer. For the S 1s, F 1s and S 2p core levels, there a single distinguishable species, as would be expected from the chemical structure, while for the C 1s there are multiple overlapping carbon signals, as expected from the structure, with the strongest around 285 eV.

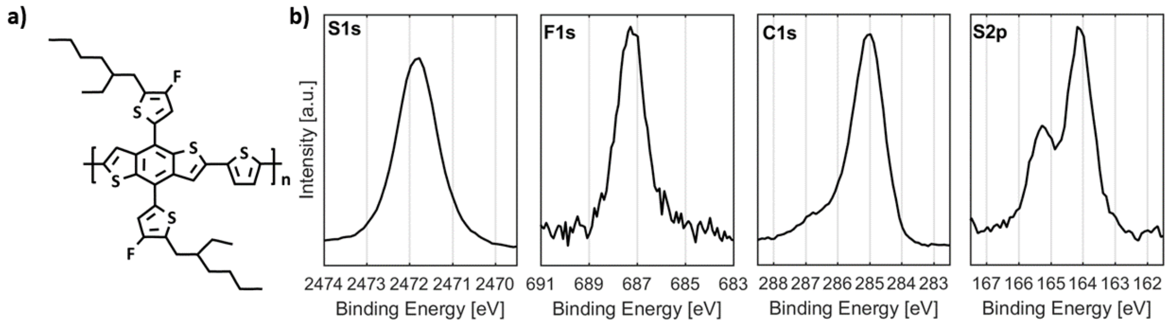

Figure S4: a) The monomer structure of P3. b) The S 1s, F 1s, C 1s and S 2p core level spectra of the P3 sample, measured at the GALAXIES beamline at SOLEIL using a photon energy of 3000 eV referenced against Au $4f_{7/2}$ at 84.0 eV.

Au/P3/$Cs_{0.17}FA_{0.83}PbI_3$ operando measurements

Figure S5 shows the Pb/Au, I/Au, C1s/Au and I/Pb ratio of the interface during open circuit and short circuit in the dark. The difference in the absolute value of the ratios is because measurements were carried out in different sample spots, which could have slightly different coverage of both the P3 polymers and Au.

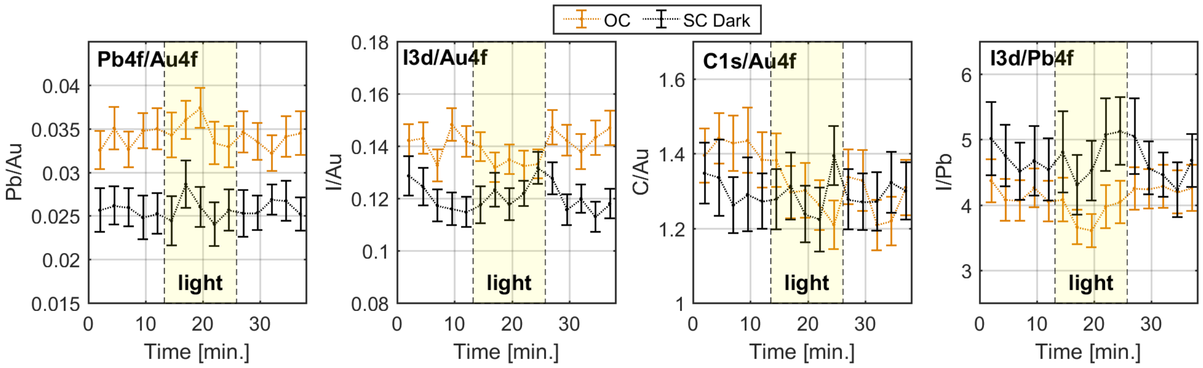

Figure S5: The Pb/Au, I/Au, C/Au and I/Pb ratio of the TiO_2/$Cs_{0.17}FA_{0.83}PbI_3$/P3/Au sample at short circuit in the dark and open circuit with and without light as a function of time. The error bars indicate 95 % confidence interval.

Core level to VBM binding energy difference

Figure S6 show the Voigt fit of Pb4$f_{7/2}$ core level and the logarithmic intersection fit of the VBM of the perovskite used in this study. Table S3 show the corresponding binding energies as well as the core level to VBM binding energy difference. The binding energy and energy scale at CoESCA was calibrated by aligning both the C 1s and S 2p core levels of the P3 sample measured at CoESCA with those measured at SOLEIL (themselves calibrated against Au 4f), shown in Figure S7. By doing this the valence band will be in the same position for all measurements. Table S4 show the position of the strongest C1s signal and the VBM as well as the C 1s to VBM binding energy difference for the P3 sample.

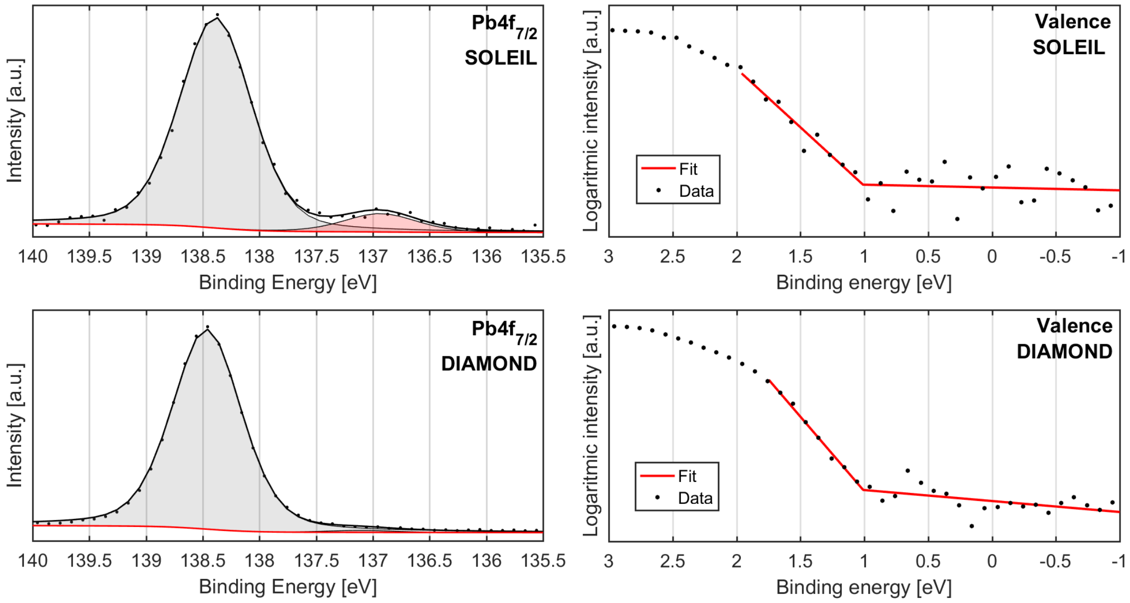

Figure S6: The fit of the I 3$d_{5/2}$ and Pb 4$f_{7/2}$ core levels and valence band edge of the different perovskite compositions. Measured at the GALAXIES beamline at the SOLEIL synchrotron using a photon energy of 3000 eV and referenced against Au 4$f_{7/2}$ at 84.0 eV.

Table S3: The binding energy of Pb $4f_{7/2}$ and I $3d_{5/2}$ core levels as derived from the Voigt fit and the valence band maximum as derived from the logarithmic intersection fit.

	SOLEIL	DIAMOND
VBM	1.01 ± 0.09	1.01 + 0.05
Pb 4f	138.39 ± 0.002	138.46 ± 0.001
I 3d	619.21 ± 0.001	619.30 ± 0.001
Pb 4f-VBM	137.38 ± 0.09	137.46 ± 0.05
I 3d-VBM	618.20 ± 0.09	618.29 ± 0.05

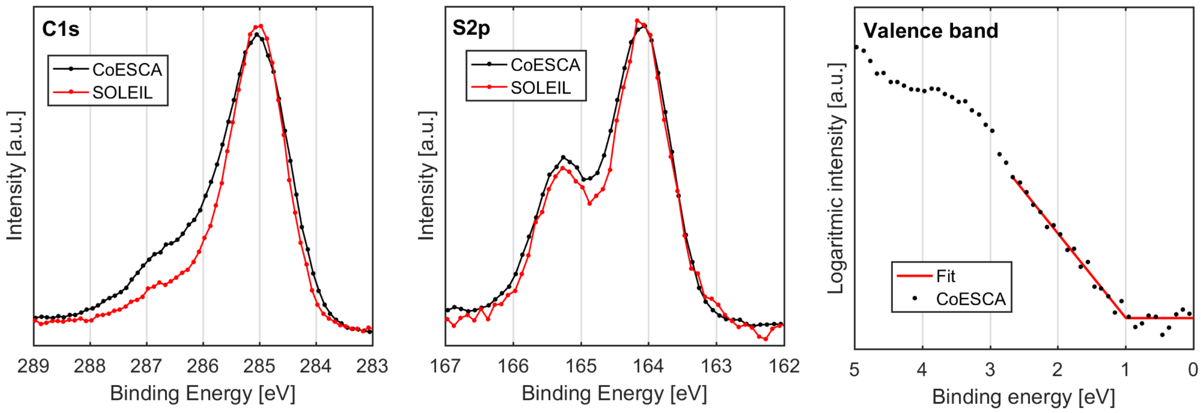

Figure S7: The C 1s, S 2p core levels and valence band of the P3 sample measured at CoESCA and SOLEIL at a photon energy of 535 eV and 3000 eV, respectively. The fit of the valence band edge of the P3 material is included.

Table S4: The binding energy of the strongest C 1s core level signal as derived from the Voigt fit and the valence band maximum as derived from the logarithmic intersection fit.

	P3
VBM	1.00 ± 0.07
C 1s	285.030 ± 0.003
Difference	284.03 ± 0.07
Band gap[3]	1.98

References

(1) Powell, C. J. Elemental Binding Energies for X-Ray Photoelectron Spectroscopy. *Appl. Surf. Sci.* **1995**, *89* (2), 141–149. DOI: 10.1016/0169-4332(95)00027-5

(2) Svanström, S.; García Fernández, A.; Sloboda, T.; Jacobsson, T. J.; Rensmo, H.; Cappel, U. B. X-Ray Stability and Degradation Mechanism of Lead Halide Perovskites and Lead Halides. *Phys. Chem. Chem. Phys.* **2021**, *23* (21), 12479–12489. DOI: 10.1039/D1CP01443A

(3) Zhang, F.; Yao, Z.; Guo, Y.; Li, Y.; Bergstrand, J.; Brett, C. J.; Cai, B.; Hajian, A.; Guo, Y.; Yang, X.; Gardner, J. M.; Widengren, J.; Roth, S. V.; Kloo, L.; Sun, L. Polymeric, Cost-Effective, Dopant-Free Hole Transport Materials for Efficient and Stable Perovskite Solar Cells. *J. Am. Chem. Soc.* **2019**, *141* (50), 19700–19707. DOI: 10.1021/jacs.9b08424
